# Transcriptome Profiling of Beach Morning Glory (*Ipomoea imperati*) under Salinity and Its Comparative Analysis with Sweetpotato

**DOI:** 10.1371/journal.pone.0147398

**Published:** 2016-02-05

**Authors:** Julio Solis, Niranjan Baisakh, Steven R. Brandt, Arthur Villordon, Don La Bonte

**Affiliations:** 1 School of Plant, Environmental, and Soil Sciences, Louisiana State University Agricultural Center, Baton Rouge, LA, United States of America; 2 Louisiana Digital Media Center, Louisiana State University, Baton Rouge, LA, United States of America; 3 Sweet Potato Research Station, Louisiana State University Agricultural Center, Chase, LA, United States of America; National Institute of Plant Genome Research, INDIA

## Abstract

The response and adaption to salt remains poorly understood for beach morning glory [*Ipomoea imperati* (Vahl) Griseb], one of a few relatives of sweetpotato, known to thrive under salty and extreme drought conditions. In order to understand the genetic mechanisms underlying salt tolerance of a Convolvulaceae member, a genome-wide transcriptome study was carried out in beach morning glory by 454 pyrosequencing. A total of 286,584 filtered reads from both salt stressed and unstressed (control) root and shoot tissues were assembled into 95,790 unigenes with an average length of 667 base pairs (bp) and N50 of 706 bp. Putative differentially expressed genes (DEGs) were identified as transcripts overrepresented under salt stressed tissues compared to the control, and were placed into metabolic pathways. Most of these DEGs were involved in stress response, membrane transport, signal transduction, transcription activity and other cellular and molecular processes. We further analyzed the gene expression of 14 candidate genes of interest for salt tolerance through quantitative reverse transcription PCR (qRT-PCR) and confirmed their differential expression under salt stress in both beach morning glory and sweetpotato. The results comparing transcripts of *I*. *imperati* against the transcriptome of other *Ipomoea* species, including sweetpotato are also presented in this study. In addition, 6,233 SSR markers were identified, and an *in silico* analysis predicted that 434 primer pairs out of 4,897 target an identifiable homologous sequence in other *Ipomoea* transcriptomes, including sweetpotato. The data generated in this study will help in understanding the basics of salt tolerance of beach morning glory and the SSR resources generated will be useful for comparative genomics studies and further enhance the path to the marker-assisted breeding of sweetpotato for salt tolerance.

## Introduction

Salt and drought stresses are two major abiotic constraints to furthering crop food production. Water soluble salt affects more than 800 million hectares of land worldwide. Salts in soil can primarily originate from soil parent material, secondarily from irrigation water, or from fertilizers, manures, compost, and other amendments [1]. Soil salinity affects sweetpotato productivity and expansion of cultivation in many parts of world, including Africa where it is a staple food [2]. A better understanding of underlying mechanism of salt tolerance in plants adapted to saline environments may offer clues to alleviate limitations to crop productivity and opening new crop lands with a saline environment.

Beach morning glory (*Ipomoea imperati*, Convolvulaceae), a native of tropical Central America and part of southeastern North America, is a prostrate vine that is distributed in the backshore of coastal beaches of most continents, and is common in the dune system (3). This species thrives in poor soils enriched in salt, and is therefore adapted to saline environments. Identifying genes for salt tolerance in this species might facilitate the usefulness of current transcriptome and genomic resources available for sweetpotato [3,4] and serve as a basis towards genic marker-assisted breeding for salt tolerance in sweetpotato. Next generation sequencing (NGS) has been utilized to unravel genes and pathways on a transcriptome-wide scale in non-model plant species For example, 454 and Illumina platforms have benefited the transcriptome analysis of sweetpotato by identifying genes involved in the development of storage roots (4) and documenting functional transcripts on a global scale [5,6]. None of these studies has focused on transcriptome and gene expression profiling of sweetpotato under salinity stress. Lack of a reference sweetpotato genome and the unknown potential existing in sweetpotato for salt tolerance is unexplored. Transcriptome profiling by next generation sequencing technologies are being widely applied in the study to identify components that mediate abiotic stress responses in plants, specifically from wild and non-model plants [7–9].

Plant adaptation to salinity depends primarily on three mechanisms: salt exclusion, osmotic stress tolerance, and the tolerance of tissue to accumulated salt ions [10]. At the molecular level, salt tolerance in plants is associated with genes implicated in ion homeostasis by transporters located in the plasma membrane and in the tonoplast [11]; efflux and sequestration of ions are the two underlying strategies by which plants can adapt to growth when challenged with salinity stress. Sodium-hydrogen (Na^+^/H^+^) exchangers and high-affinity potassium (K^+^) transporters (HKT) that are stimulated in response to an increase in sodium ions are among key components associated with salinity tolerance. The *Arabidopsis thaliana* vacuolar *AtNHX1* transporter [12] and the membrane SOS1 (salt overly sensitive 1) transporter [13] are some of the most studied proton-transporters that confer salt tolerance in *Arabidopsis* and in other plants [14]. Sequestration of sodium in vacuoles catalyzed by vacuolar Na^+^/H^+^ antiporters requires a transmembrane electrochemical potential, so it is not surprising that genes encoding vacuolar H^+^-ATPase (V-ATPase) and H^+^-pyrophosphatase (H^+^-PPase), which generate this membrane potential, are found to enhance salinity tolerance [15–17]. The plasma membrane Na^+^/H^+^ antiporters of SOS1 family have been implicated in Na^+^efflux and the members of HKT1 are responsible for the influx and redistribution of Na^+^ from shoots to roots [10,18]. The signal transduction networks activated in response to salt stress involve components of abscisic acid (ABA) signaling, plant mitogen-activated protein kinase (MAPK), calcium-dependent protein kinase (CDPK) and the salt overly sensitive (SOS) pathways. The SOS pathway is key to regulating Na^+^/K^+^ ion homeostasis and SOS1-mediated salt tolerance in plants [19–21]. SOS3 and SO2 proteins, encoding calcineurin B-like proteins (CBLs) and CBL-interacting protein kinases (CIPKs) respectively, are important in the calcium signaling pathway that is used by plants in response to environmental cues through post-translational modifications [22]. It is recognized that cross-talking at different levels of these pathways exists in response to salt, dehydration, drought and cold tolerance [23]. ABA signaling is known to play a critical role in the plant response to salinity and ABA-mutants perform poorly under salt stress [24]. The up-regulation of *AtNHX1* for salt tolerance requires the synthesis of ABA [24].

The role of the transporters in the physiology of plant, in salt stress tolerance, and developmental processes is complex. For instance, in *Ipomoea tricolor* a vacuolar Na^+^/H^+^ exchanger with similarity to the *AtNHX1* gene, was found regulating the vacuole pH [25] and associated with flower coloration; a similar antiporter has been reported for *I*. *nil* [26]. It is expected that these transporters have additional roles besides salt-tolerance given the abundance of genes for vacuolar transportation in salt-sensitive plants. Moreover, Na^+^ sensing in plants appears to have evolved by generating natural variants of the *SOS1* gene that confers different adaptation to saline environments in the carrier [21,27], and their roles appear to contribute to different responses to salt stress besides ion homeostasis [28]. SOS signaling events ultimately trigger the transcription of multiple stress-responsive genes. Identification of master genes implicated in the translocation of signaling events in the upstream pathway to *SOS1*-like transporters and in the *SOS1* downstream targets, all of which might be relevant genes for plant salt-stress tolerance, could be enhanced by transcriptome analysis. Although, cross-talking of the signaling mechanism for most abiotic stresses might impede an immediate use of any master regulatory gene, identification of salt-responsive genes from salt-tolerant species represent an efficient approach [6,29]. Transcription factors (TF) are the most powerful candidates to enhance salt tolerance in plants, as overexpression of a TF can lead to up-regulation of a whole array of genes under its control [30]. However, gene synergism other than TFs such as the observed interaction of AtNHX1 and the vacuolar ATPase (V-ATPase) [15] represent a strategy to enhance salt stress tolerance in plants. Likewise, overexpression of wheat *TNX1* antiporter and the H^+^-pyrophosphatase *TVP1* improved salt and drought stresses tolerance in *Arabidopsis* [31]. Two different types of genes, *IbNFU1* for iron sulfur cluster machinery [32] and a gene pyrroline-5-carboxylate reductase (*IbP5CR*) for proline metabolism [33] were identified in sweetpotato for salt tolerance. *IbNFU1* was shown to enhance salt tolerance through mechanisms for proline accumulation by protecting membrane integrity and photosynthesis, and by ROS scavenging [34].

The objective of the present study was to characterize the transcriptome of beach morning glory and identify its tissue-specific salt responsive genes. The results of comparative analysis against other *Ipomoea* transcriptome datasets are discussed that may open up a forum for strategies for sweetpotato breeding for salt tolerance.

## Materials and Methods

### 2.1. Biological material and treatments

No specific permissions were required for collection of beach morning glory from coastal Louisiana (Holly Beach, N 29°45' 38.588", W 93° 34' 9.365"), which researchers have free access to and the study did not involve any endangered or protected species. The experiment was carried out inside the university greenhouse. Plants of beach morning glory were collected with sandy soil attached to the roots from the coastal areas of Louisiana and grown inside greenhouse conditions at a day/night temperature regime of 29/22°C and 14 h day light. From a pilot-scale salt stress experiment, we observed stress symptoms after three days of salinity treatment at 300 mM NaCl (S1 Fig). Therefore, 300 mM NaCl was set as the experimental threshold for this study. After a month of acclimation, 12 plants were subjected to salinity (300 mM NaCl) and three plants were watered without salt, as control. Shoot (leaf and stem) and root tissues were taken at 0 h (control), 1 h, 24 h, 72 h, and 1 week of salt treatment for gene expression studies. Each treatment had three biological replications. Tissues were frozen in liquid nitrogen and stored at -80°C until processing for RNA extraction.

Vegetative cuttings of ‘Beauregard’ sweetpotato were transplanted in dry sand in cylindrical tubes (50 cm x 9.82 cm) under greenhouse conditions similar to the one discussed above and watered every three days for two weeks. Plants were subjected to 500 ml of 150 mM NaCl after 2 weeks and total roots and leaves were sampled at 24 h and 72 h of stress. Likewise, non-stressed plants tissues were sampled at each time point as control. Tissues were frozen in liquid nitrogen and stored at -80°C until processing for RNA extraction. All samples were done in triplicate.

### 2.2. RNA extraction and library construction

Total RNA was extracted with Trizol reagent (Invitrogen, Carlsbad, CA) according to the manufacturer’s instructions. Aliquots of all individual RNA samples (~ 4 μg) from roots or shoots under salt stress were pooled prior to sequencing and RNA (~16 μg) of both control root and control shoot samples were kept separate. Four 454 libraries were constructed by MOgene LLC (St Louis, MO) from control shoot (CS), control root (CR), pool of salt-stressed root (SR), and pool of salt-stressed shoot (SS).

### 2.3. Sequence assembly and analysis

A reference transcriptome was produced by combining all the de-multiplexed reads from all libraries with the MID index removed (as given by MOgene LC). All raw data were further cleaned and filtered by removing low-complexity, organellar, dust, and short reads (<100 nt) using SeqClean. The reference transcriptome (consensus transcripts and singlets) was generated with iAssembler [35]. Output was further annotated and analyzed for marker sequences as described in next sections. Raw reads used in this work will be available in SRA database of NCBI.

BLASTN [36] was used for comparative analysis of the unigenes against transcripts from related species of the Convolvulaceae family. Transcriptome data sets from four species were considered: the sweetpotato gene index of root transcriptome (3), called “PBL assembly”; a custom unpublished sweetpotato assembly called “CAP3 assembly”, which integrates all expressed sequence tags deposited at ESTdb of NCBI, and all reads from two independent sweetpotato transcriptome libraries from stem and leaves (Dr. R. Schafleitner, personal communication) and from root libraries (personal communication with Dr. N. Firon, 2011); a sweetpotato transcriptome deposited at the Transcript Shotgun Assembly (TSA) database, called “sweetpotato TSA assembly” (4); The Gene Index of morning glory (*I*. *nil*) by Dana Faber Cancer Institute (DFCI), released on July 1, 2008 [37]; and The TIGR Plant Transcript Assemblies [38] of *I*. *trifida* (accessed on July 10, 2007. The identifiers (accession number, header) of each sequence of the above databases were added as a prefix to indicate the source: IbPBL, IbCAP3, IbTSA, In, It, for PBL, CAP3, TSA, DFCI and TIGR assemblies, respectively. In addition, the *I*. *purpurea* deposited in the TSAdb under accession number GALY01000000 (sequences GALY01000001-GALY01086691) were included and the prefix Ip was added to each accession entry in our analysis. Likewise, a suffix (_RC) to each entry identifier of each sequence was added to indicate that the reverse complementary sequence was used in our analysis. Raw reads from each sweetpotato library can be requested from appropriate authors [3,39] and our custom sweetpotato databases are available upon request.

### 2.4. Mining for microsatellites

*In silico* analysis was performed on the transcriptome of beach morning glory to identify microsatellites/SSR (simple sequence repeats,) y using the SSR Locator tool [40]. Repeats were searched on criteria that a dinucleotide or trinucleotide repeat should appear at least six and five times, respectively, and tetra, penta and hexa nucleotide repeats should appear five times each. Primer pairs flanking each SSR were designed using the integrated Primer3 package. Virtual-PCR analysis was conducted to map SSR primers on sequences of custom databases of four *Ipomoea* species described before to investigate the ability of the primers to amplify potential homologous targets across the *Ipomoea* genus.

### 2.5. Annotation and identification of transcripts associated with salt stress

The consensus transcripts and singlets (at least 100 bp), hereafter mentioned as unigenes, were queried to the Uniref90 protein database using BLASTX [36] with an e-value cut-off of 1e-06. The top 20 hits were assigned to each unigene and cross-referenced to NCBI database to further annotate and to obtain the relevant Gene Ontology (GO) term. A custom TAIR10 database [41] was used for querying *Arabidopsis thaliana* gene indices.

To identify candidate transcripts associated with salt stress response, all raw reads that were mapped in each consensus sequence were processed by custom Perl scripts. Counting number of reads from each CS, CR, SR and SS libraries contained in each unigene were used as an indicator of their association with salt stress responses (S1 Table). As criteria, a unigene with 0 read count from both CS and CR and with at least 2 reads from both/either of SR and SS libraries was putatively identified as a gene associated with salt stress response. This was an arbitrary relative threshold established to estimate whether a read was overrepresented in a library. Only unigenes with well-supported annotation and abundance of reads from salt-stress treatments were considered for further analysis.

### 2.6. Expression analysis by quantitative real time PCR (qRT-PCR)

Reverse Transcription PCR and quantitative real time PCR (qRT-PCR) was used to study the expression of selected genes in response to salt stress at different time points in beach morning glory (leaf only at 1 h, 24 h, 72 h, and 168 h) and sweetpotato (leaf and root at 24 h and 72 h). First strand cDNA synthesis was performed by reverse transcription of 1 μg total RNA using the iScript cDNA synthesis kit (Bio-Rad, Hercules, CA) as per manufacturer’s instructions. Then, qRT-PCR reactions were performed with 2 μl of diluted cDNA (1:3) on a MyiQ Real-Time PCR detection system (Bio-Rad, Hercules, CA) using iQ SYBR Green supermix (Bio-Rad, Hercules, CA) in a final volume of 20 μl following the recipe and thermal profile as described earlier (42). Each reaction was performed in triplicate (three independent plants—biological replicates), and the average threshold cycle (Ct) was used to estimate the relative expression of each transcript to control samples normalized against the endogenous reference gene elongation factor-1-alpha (IbElf-1α) as previously reported [42].

## Results and Discussion

Adapted to grow in coastal areas and a close relative of hexaploid sweetpotato, beach morning glory (*Ipomoea imperati)* represents a unique genomic resource for salt tolerance in sweetpotato and related crops. The ability to further abiotic stress breeding in sweetpotato is now plausible given the availability of NGS as a low-cost, large-scale approach for transcriptome sequencing [43]; as a result an increased knowledge of functional genes has been identified for sweetpotato [3–5,39]. However, little information has been derived from the previous reports on the changes in its transcriptome in response to abiotic stress; to our knowledge all existing transcriptome libraries developed were from tissues at different developmental stages except for a single library from drought stressed leaves [39]. We conducted transcriptome profiling by 454 sequencing of beach morning glory under salt stress to dissect salt tolerance mechanisms at the transcriptional level. The raw sequence reads are publically available at the NCBI SRA (http://ncbi.nlm.nih.gov/sra) under the accession number SRP066755. The results presented in this research may benefit sweetpotato breeding for tolerance to salinity.

### 3.1. *De Novo* Transcriptome Assembly

Raw reads of the pyrosequencing libraries were processed by filtering out short reads (<100nt), dust sequences (consisting mostly of low complex sequences), and removing sequences derived from plastid, mitochondria, and rRNA. Removal of contaminants in sequencing input (reads) has improved quality of *de novo* assemblies [39] and common when working with high-throughput sequencing data [44]. The workflow implemented for the pipeline of iAssembler requires that contaminants from plastid and rRNA be removed before analysis to avoid misassemblies and misinterpretations [35]. Statistics of raw and filtered sequence data from each library are presented in Table 1. As expected, sequence reads from shoot libraries were highly enriched in plastid sequences. Average length of filtered reads ranged from 519.7 bp to 538.8 bp. After removing 98,402 and 100,396 reads as short size/low complex sequences and contaminants, respectively, 485,520 reads comprising 151,942,782 bases were used to develop a reference transcriptome.

**Table 1:** Summary of statistics of filtered and unfiltered reads from libraries of shoot (S) and root (R) tissues of beach morning glory under control (C) and salt stress (S).

	CS	SS	CR	SR	Combined libraries
Number of reads in filtered data sets	49,079	44,442	88,921	104,142	286,584
Total length (nt) in filtered data sets	26,383,034	23,527,656	47,906,340	54,125,752	151,942,782
Mean length (bp) in filtered data sets	537.6	529.4	538.8	519.7	530.2
Maximum length (bp) in filtered data sets	1079	1029	1231	1048	1231
Median length (bp) in filtered data sets	581	574	580	566	574
Number of reads before filtering	96,102	119,923	118,410	151,085	485,520
Number of reads filtered due to short size (100nt) or low complexity	18,224	22,810	23,470	33,898	98,402
Number of reads filtered as being contaminant (rRNA, organellar)	28,756	52,639	5,987	13,014	100,396

CS = control shoot; SS = salt stressed shoot; CR = control root; SR = salt stressed root.

**Fig 1:**
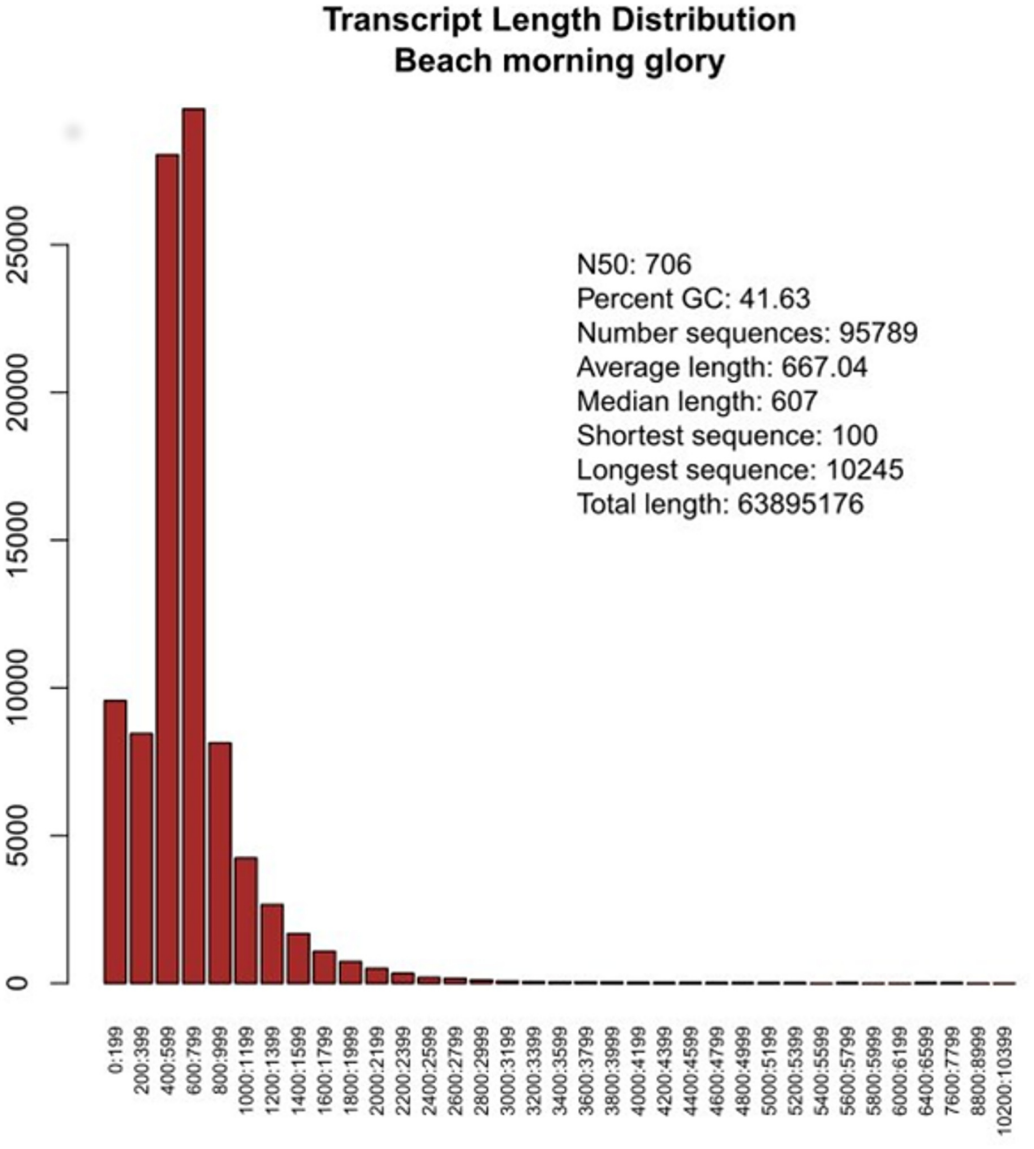
Summary and distribution of unigenes from beach morning glory.

A unique assembly combining all reads from the four libraries were developed using the iAssembler package, which represented the first gene index for beach morning glory. Sequence assembly resulted in 95,790 unigenes comprising 32,291 contigs and 63,499 singlets (Fig 1). Size of contigs ranged from 102 bp to 10,245 bp whereas that of singlets ranged from 100 bp to 1,231 bp. Annotation of the beach morning glory transcripts revealed that 34,053 of out of 95,790 unigenes did not have matching sequences in the Uniref90 database (S1 and S2 Tables). The unannotated sequences comprised 5,902 contigs and 28,151 singlets, which may represent new genes and/or non-coding regions and could be unique to the *Ipomoea* genus, although their number is less compared to previous studies in sweetpotato [44]. A large fraction of the transcripts (47,602 out of 95,790 transcripts) lacked a putative orthologous sequence in the model plant *Arabidopsis thaliana* (S3 Table). This emphasizes the need of developing genomic resources for Convolvulaceae order. Further detail of this transcriptome assembly and the aligned reads are in the supplementary file in SAM-format (S1 Text).

### 3.2. Identification and expression analysis of candidate salt-tolerance genes in beach morning glory

A number of candidate salt responsive genes were identified in response to salt stress in beach morning glory transcriptome (Table 2). Orthologs of these genes implicated in abiotic stress responses, including salinity, have been reported in several other crops. A large number (4,045) transcripts from shoot and/or salt stressed libraries (S4 Table) represent a set of candidate genes that could be unique in beach morning glory that possibly play important roles in its ability to thrive in saline soils. Comparative transcriptome analysis revealed that about half of the *I*. *imperati* transcripts (46,017 out of 95,790) have homology in sweetpotato (S5 Table). In addition, 46,215 transcripts did not have matches in the available transcriptome of four *Ipomoea* species (S5 Table). Transcripts belonging to the biological processes ‘photosynthesis’ (GO: 0015979) and ‘generation of precursor metabolites and energy’ (GO: 0006091) were highly represented in beach morning glory transcriptome (S6 Table),

**Table 2:** Candidate genes associated with salt-stress response in beach morning glory.

Unigene ID (consensus transcript, singlets)	Length (bp)	Annotation	Source of reads included consensus transcript	Transcriptome libraries of beach morning glory in which reads are overrepresented	Sweetpotato entry
UN08046	976	DRE-binding transcription factor swDREB1	Shoots and Roots	Shoots and Roots under salt stress	IbCAP3Contig720.1
UN07119	723	DREB protein (Fragment)	Shoots and Roots	Shoots and Roots under salt stress	NO DATA
UN22967	570	Ethylene responsive factor	Roots	Roots under salt stress	RT_307632.1
UN14336	1063	WRKY transcription factor	Shoots and Roots	Shoots and Roots under salt stress	IbPBL_S_PBL_c6924
UN14262	1070	Putative cytochrome C oxidase subunit II family protein	Shoots and Roots	Shoots and Roots under salt stress	IbCAP3Contig26623.1
UN15532	710	WRKY transcription factor	Roots	Roots under salt stress	IbCAP3Contig31286.1
UN06652	528	sodium transporter HKT1-like	Shoots and Roots	Shoots and Roots under salt stress	IbCAP3Contig22603.1
UN07231	1433	Inorganic pyrophosphatase (PPase)	Shoots and Roots	Roots under salt stress	IbCAP3Contig23069.1
UN31159	1043	soluble inorganic pyrophosphatase-like (PPase)	Shoots and Roots	Shoots and Roots under salt stress	IbCAP3Contig22172.1
UN25947	630	Na^+^/H^+^ antiporter	Shoots and Roots	Shoots under salt stress	NO DATA
UN26363	822	Cation:cation antiporter	Shoots and Roots	Roots under salt stress	IbPBL_S_PBL_c17537 IbCAP3Contig6212.1
UN18353	1000	Osmotin-like protein	Shoots and Roots	Roots under salt stress	IbPBL_S_PBL_lrc38257 IbCAP3Contig10297.1
UN04545	1033	CBL-interacting protein kinase	Shoots and Roots	Roots under salt stress	IbPBL_S_PBL_c3473
UN20155	1518	Calcineurin B-like protein	Shoots and Roots	Roots under salt stress and in shoots	IbnuEST_JG699772.1
UN04785	1349	CBL-interacting protein kinase		Roots under salt stress	IbTSA_JP111514.1
UN14651	985	CBL-interacting protein kinase		Roots under salt stress	IbTSA_JP112284.1
UN19201	594	CBL-interacting protein kinase		Roots under salt stress	IbPBL_S_PBL_c12091
UN19788	815	CBL-interacting protein kinase		Shoots under stress	IbTSA_JP106495.1
UN26778	1104	CBL-interacting protein kinase		Roots under salt stress	IbTSA_JP120118.1
UN71496	131	CBL-interacting protein kinase		Roots under salt stress	IbTSA_JP113068.1
UN74114	821	CBL-interacting protein kinase		Roots under salt stress	IbTSA_JP107110.1
UN75513	686	CBL-interacting protein kinase		Roots under salt stress	IbTSA_JP106495.1
UN83272	504	CBL-interacting protein kinase		Roots under salt stress	IbPBL_S_PBL_c10086
UN83471	468	CBL-interacting protein kinase		Roots under salt stress	IbPBL_S_PBL_lrc30596
UN84314	577	CBL-interacting protein kinase		Roots under salt stress	IbPBL_S_PBL_lrc54022
UN84911	501	CBL-interacting protein kinase		Roots under salt stress	IbTSA_JP111514.1
UN84921	323	CBL-interacting protein kinase		Roots under salt stress	IbTSA_JP140418.1
UN85200	851	CBL-interacting protein kinase		Roots under salt stress	IbPBL_S_PBL_c292
UN89027	272	CBL-interacting protein kinase		Shoots under stress	No matching hit found
UN90198	584	CBL-interacting protein kinase		Shoots under stress	IbTSA_JP121101.1
UN91252	624	CBL-interacting protein kinase		Shoots under stress	IbTSA_JP107110.1
UN94341	160	CBL-interacting protein kinase		Shoots under stress	IbTSA_JP106970.1

#### Transcription factors associated with salt stress response

Four consensus transcripts encoding a *WRKY* transcription factor (UN15532, UN14336), a cation:cation antiporter (UN26363), and an ethylene responsive factor (UN22967) were preferentially represented in the root transcriptome libraries from both beach morning glory and sweetpotato (Table 2). Members of *WRKY* transcription factors have recently been found involved in salt tolerance of plants [45–47]. S_PBL_c6924, a root-derived sweetpotato transcript (3), is an ortholog of *Arabidopsis WRKY53* (At4g23810) and matches UN14336; At4g23810-like transcripts are reported to accumulate in roots of *Medicago truncatula* under salt stress [48]. There is accumulating evidence that components of ethylene signaling are involved in salt stress and other abiotic stresses tolerance. In addition, it is well known that abiotic stress enhances the expression of members of DREB family genes. Two *DREB* transcripts (UN07119 and UN08046) were found enriched under salt stress (Table 2). Our comparative analysis between beach morning glory and sweetpotato transcriptomes revealed that UN07119 apparently lacks a homolog in sweetpotato. Ethylene response factors enhanced salt tolerance in plants [49,50], including transgenic plants [50–52]. Interestingly, two sweetpotato ERF genes, *IbERF1* and *IbERF2*, appear to be key for controlling up-regulation of various defense genes involved in abiotic stress tolerance and pathogenic resistance [53]. *IbERF1* and *IbERF2* expression was found induced in leaves within 2 hours of treatment with 100 mM NaCl; it was also up-regulated under dehydration-, chemical-, and pathogenic-stress treatments.

#### Cation transporters, membrane-associated proteins and vacuolar proteins under salt stress

The transcripts of beach morning glory that did not have close matching sequences in sweetpotato included a sodium/hydrogen (Na^+^/H^+^) antiporter (UN25947; salt stress responsive gene 13, SS13), which is known to be involved in salt tolerance in many plants. Thus transcripts coding for these antiporters may play important roles for allowing the growth of beach morning glory in saline environments. The *HKT1* (high-affinity potassium transporter) is the most promising gene identified for salt tolerance in a diverse plant species [47,54] and was also found overrepresented in the libraries from salt stressed tissues in the present study (transcript UN06652 in Table 2). Osmotin and osmotin-like proteins are a subgroup of plant defense proteins, termed PR-5, which are responsive to biotic and abiotic stress [55,56]. Osmotin and other closely related proteins are encoded by intron-less genes within a small gene family as reported for flax, potato, and tobacco. The transcript encoding an osmotin-like protein identified from beach morning glory (UN18353; SS3 in Table 3) is another example of a candidate gene for salt tolerance which was derived from root libraries under salt stress; comparative analysis of this transcript against all three custom sweetpotato transcriptomes suggested that the sweetpotato orthologs are also expressed in root. Little is known about the osmotin gene family relating to salt stress tolerance, although the first plant osmotin gene was originally isolated from salt-adapted tobacco cells [57]. Recently osmotin was found to confer tolerance for both salinity and drought [55]. Osmotin has long been recognized to accumulate into vacuolar inclusions under salt stress and confer salt tolerance to tobacco cells [58,59], suggesting that osmotin could be involved in the maintenance of high ion concentrations in the vacuole. It has also been reported that in the whole plant, the highest level of accumulation of osmotin under stress occurs in the roots [57].The closest sequence of UN93566 identified from the sweetpotato root transcriptome (S_PBL_c6736) (3) is annotated with homology to At1g36980 from *Arabidopsis* and *TaSC*, *a* an ortholog of At1g36980, was identified in a salt-tolerant wheat line that accumulated in the plasma membrane [60]. In the same study, *TaSc* was found to confer higher germination rates and seedling root length, and increased salt-tolerance of *Arabidopsis* overexpressers under salt stress by increasing the K ^+^/Na ^+^ ratio.

**Table 3:** Salt-induced transcripts used for expression analysis under salt stress in beach morning glory.

UnigeneID (Strand) [Name]*	**Forward primer**	**Reverse primer**	**Expected product size (bp)**	**Entry in sweetpotato**	**Description**
UN05312(-) [SS1]	aggcccctctctgtgatatctg	tcttgagaccttaaactgggaaca	243	IbTSA_JP110071.1	probable salt tolerance-like protein At1g75540-like
UN06652(-) [SS2]	ttgtggttcatattcttggctct	catgttttcatttgtggggaca	167	IbTSA_JP108441.1	Sodium transporter hkt1-like protein
UN18353(-) [SS3]	caccttcggaggacaacaata	cagtagatccagcagggcaac	149	IbTSA_JP134767.1	Osmotin-like protein
UN08712(-) [SS4]	ccttgcatcagatggcttatggg	cactcacgtcgtcaaaagagcc	158	IbTSA_JP112840.1	protein phosphatase 2C 25-like
UN90868(^+^) [SS5]	tcttcatccttggggaagtcac	tccaagaaattcatccagctgcca	222	IbTSA_JP108274.1	salt tolerance-like protein At1g78600-like
UN25202(^+^) [SS6]	ttgagtttccgggagataaagc	cattttattctccctcttggcatg	171	IbTSA_JP115696.1	salt tolerance-like protein At1g78600-like/Zinc finger protein CONSTANS-like protein
UN93566(-) [SS7]	gtcgtttgcagcgccgtcaaa	tcaatctccattcgccttcctcata	139	IbPBL_S_PBL_c6736	transmembrane protein 50a, putative [*Jatropha curcas*]
UN85241(-) [SS8]	tacttgctgggcctggagtg	caaatttgttttccagctccagt	170	IbPBL_GM0Z85L06HJZAC	Na^+^/H^+^ antiporter (sos1, salt overly sensitive 1)
UN15396(+) [SS9]	cattccctgcatgttaagaacct	tatttccaggcattgtttggatg	206	IbTSA_JP104644.1	DNA binding protein
UN07231(+) [SS10]	gtgctcttgtcatgctcactcc	gccaacagtgtcaccaatcaca	243	IbTSA_JP106494.1	Inorganic pyrophosphatase
UN05798(+) [SS11]	caagaaaatcttggccatgcagc	cagcctccaatttgccacgaatt	162	IbTSA_JP121852.1	*Nicotiana attenuata* jasmonate ZIM domain protein h mRNA; *Solanum lycopersicum* salt responsive protein 1 (SRG1)
UN04483(+) [SS12]	cgtggctgaaaactcacctctc	atgcgcccataagttcatcgagc	166	IbTSA_JP108229.1	Arginine-aspartate-rich RNA binding protein-like
UN29547(-) [SS13]	gtcagccaccagtaattgatgt	gcccaactattgccaagacttac	206	IbTSA_JP113383.1	salt tolerance protein 5-like protein [*Solanum tuberosum*]
UN05755(-) [SS14]	tgatacccgcgacttcaagatt	gctctcaatcacaacagcaaca	226	IbTSA_JP120116.1	Voltage-dependent calcium channel protein TPC1A
UN17963	ccaagattgatagacggtctgg	ctcatgtccctcacagcaaaac	160	IbTSA_JP106582.1	Elongation factor 1-alpha

*A plus sign next to unigene identifier means that coding sequence is the given sequence, and a minus sign means that coding sequence is in reverse complementary strand.

Beach morning glory is a relative of sweetpotato and exhibits a high degree of salt tolerance. In contrast to other plants, it does not have salt glands in its leaves. Thus, mechanisms that help to sequester the excessive Na^+^ into the vacuoles may be the predominant adaption mechanism to cope with high salinity. In higher plants, salt extrusion from the cell and the salt sequestration into vacuoles are carried out by antiporters, which require the energy dependent proton pumps [P-type H^+^-ATPase, V-type H^+^ ATPase and V-type H^+^- pyrophosphatases (V- PPases)]. Genes encoding V-PPases are among well-studied candidate genes for salt tolerance in halophyte plants, and consequently used for conferring salt and drought tolerance by overexpression in model as well as non-model plants [16,17,29,31,61]. In this study, two V-PPases (UN07231, UN31159; Table 2) were overrepresented in both shoot and root libraries under salt stress. It is documented that all the major processes such as photosynthesis, protein synthesis and energy and lipid metabolism are affected during the onset and development of salt stress within a plant [62]. Indeed, we have found transcripts related to genes in these processes in different amounts from each of the *I*. *imperati s*hoot and root libraries under both control and stressed conditions.

#### Genes in signal transduction pathways in response to salt stress in beach morning glory

Many salt-tolerance related genes act via signaling pathways. Calcineurin B-like (CBL)-interacting protein kinases (CIPKs) transcripts were found enriched in both shoot and root tissues under salt stress (Table 2). All the *CIPK* transcripts except UN89027 had a matching transcript in at least one of sweetpotato transcriptomes. Transcript UN14651, encoding a CIPK, had high reads in salt stressed tissues and matched to the sweetpotato root-derived transcript S_PBL_c574 annotated as SOS3 (SOS3-interacting protein 3, At4g30960). SOS3 is a paradigmatic example of a CIPK involved in mediating salt tolerance and developmental processes in *Arabidopsis*. Our study revealed that three CIPKs (UN04785, UN19201, UN84911) had a preferential expression in root tissues of sweetpotato; our comparative analysis against two sweetpotato transcriptomes found that the putative sweetpotato orthologs of these CIPKs were identified as overrepresented in roots [2,3]. The sweetpotato transcript S_PBL_c3473 (3), identified to be orthologous to beach morning glory entry UN84911, matched with *AtCIPK23* (*At1g30270*), known to be involved in activating a plant potassium channel in concert with other calcineurin B-like calcium sensors (CBLs). Phosphorylation of CBLs by their interacting CIPKs has been shown to be required for full activity of CBL-CIPK complexes toward their target proteins [19,63,64]. New CIPKs participating in reduced shoot Na^+^ accumulation and retention of K^+^ under salt stress conditions are being discovered in other species, with at least four CIPK genes reported in *Arabidopsis* [65], thus underscoring the importance of *CIPK* genes for salinity tolerance in plants [66–68]. In higher plants, the CBL-CIPK network typically consists of about 10 CBLs and 25–30 CIPKs [22]. The present study showed multiple CIPKs found in beach morning glory transcriptome. Furthermore, there is a crosstalk between the CBL-CIPK pathway, the low-K⁺ response pathway, the ABA signaling pathway, the nitrate sensing and other signaling pathways [23]. Potassium is an essential macronutrient for plants and mechanisms that alter its homeostasis, such as salt stress due to NaCl, undoubtedly trigger other adaptive mechanisms to maintain uptake of K^+^ inside the cells. Plant K^+^ acquisition and homeostasis is driven by K^+^-channels and their low and high affinity for K^+^ depends on the phosphorylation of the transporters by CIPKs and other sensors. Since plants absorb K^+^ from soils through root cells, the altered expression of CIPKs in beach morning glory root might be related to K^+^-transport. Studies involving *TaSC* gene indicated that it may involve the CDPK pathway [60], with CDPKs known to be in pathways that lead to enhanced expression of the known salt-tolerant genes such as *AtCOR15a*, *AtRD29B*, *AtP5CS1*, and *AtADH* [69].

#### Validation of expression of salt-responsive genes

A set of 14 transcripts, from different categories and overrepresented as either members of GO term categories or transcripts enriched in the libraries in response to salt stress, were selected for validation of their expression corresponding to the results of the transcriptome sequencing analysis. Detailed description of these transcripts and primers are presented in Table 3.

**Fig 2:**
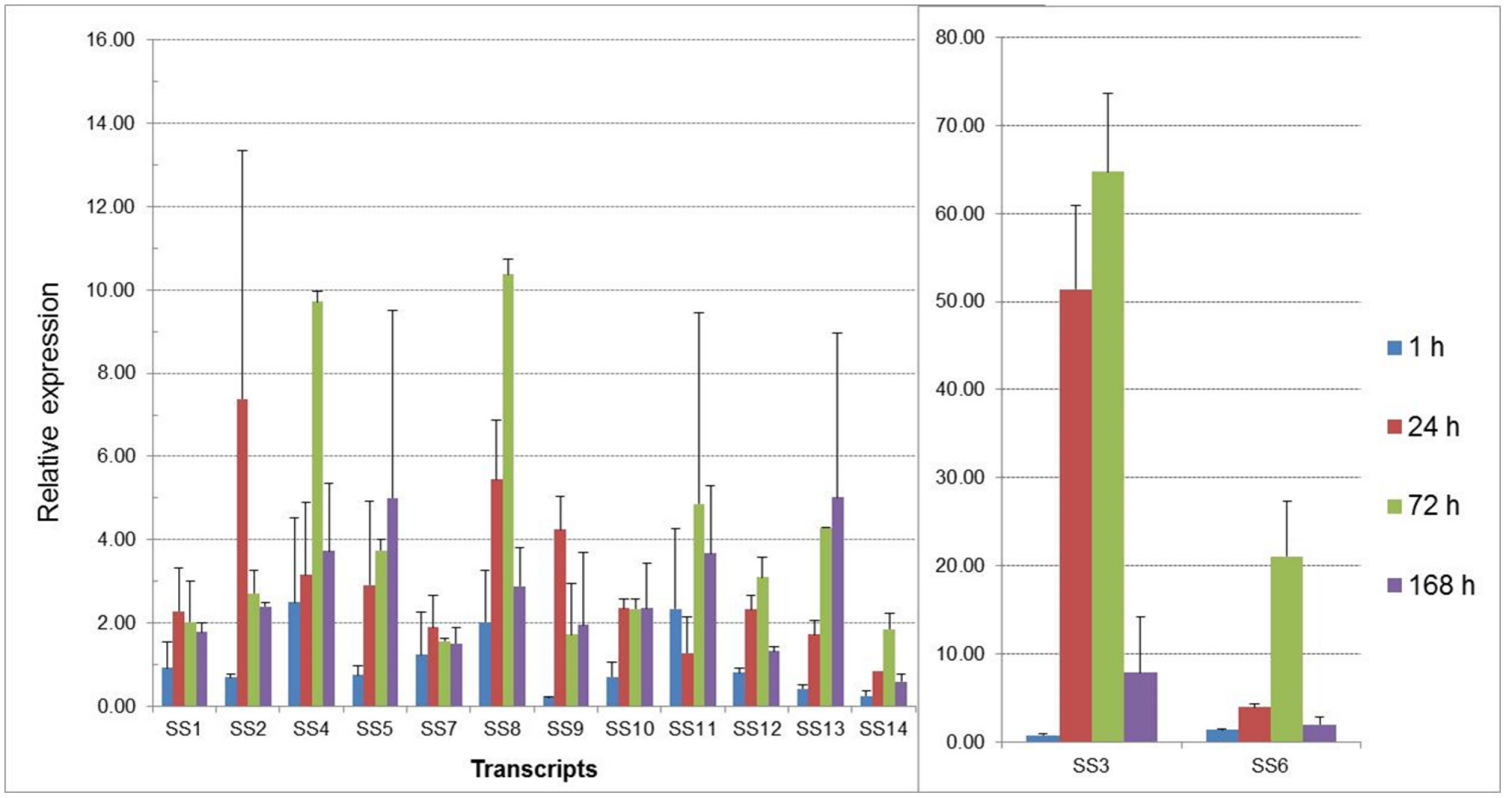
Relative expression of selected genes at different time points of salt stress in shoot tissue of beach morning glory. Salt stress (SS) responsive transcript names are shown in Table 3.

Expression analysis suggested that genes involved in salt response in beach morning glory contrasted with their response in sweetpotato (Fig 2). Transcripts coding for sodium transporter hkt1-like protein (UN06652; salt stress responsible 2, SS2 gene)), protein phosphatase 2C 25-like (UN08712; SS4), Na^+^/H^+^ antiporter (UN85241; SS8), and salt tolerance protein 5-like protein (UN29547; SS13) were consistently up-regulated (2 to10 fold) in response to salt stress in shoot tissues at early and late stages of salt imposition. Although, nine out of 14 transcripts did not show enhanced expression after 1 hour of stress, their expression was up-regulated in the shoots of beach morning glory at 24 h and 1 week of stress. All 14 transcripts except for the voltage-dependent calcium channel protein (UN05755; SS14)) were enhanced under salt stress supporting their role in salinity tolerance. In addition, the inconsistency in expression level observed in shoot of beach morning glory for osmotin-like protein (UN18353; SS3) could be due to the specificity of the primers and RT PCR conditions.

The results indicated that ion homeostasis by the regulation of transporters appear as a predominant mechanism of salt adaptation of *I*. *imperati*. Two Na^+^/H^+^ antiporters such as the vacuolar transporter NHX1 and the plasma membrane transporter SOS1 were the first components identified in *Arabidopsis* to be involved in mechanism of Na^+^ detoxification in cells [12,13], and it was their up-regulation under salt stress that provided supporting evidence of their role in salt tolerance. We also found similar transporters in beach morning glory transcriptome. *NHX1*-, *SOS*- and *HKT1*-like genes are found in both salt-tolerant and salt-sensitive plants [10], although their efficiency and underlying responses may be unique to each species [21,27]. Salt-stress signaling in plants occurs via abscisic acid-dependent and-independent pathways, and several transcription factors. Identification of components downstream of NHX1-like, SOS-like, and HTK1-like Na^+^ transporters in beach morning glory is needed. A new mechanism of 14-3-3 proteins-mediated regulation of SOS components has been found in plants [70]. We expect that, given the uniqueness of beach morning glory, further investigation of the current transcriptome will contribute to novel mechanisms of regulation of ion transport under salinity.

**Fig 3:**
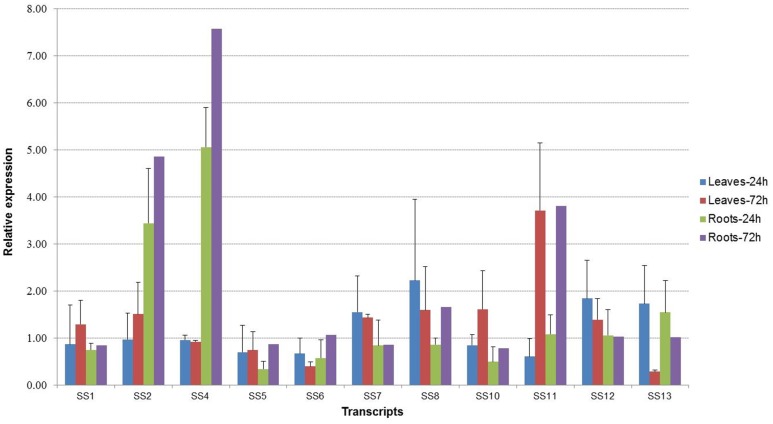
Relative expression of selected genes at 24 and 72h of salt stress in leaf and root tissues of sweetpotato. Salt stress (SS) responsive transcript names are shown in Table 3.

Species-specific differences were observed when comparing expression of the selected transcripts between beach morning glory (Fig 2) and sweetpotato (Fig 3). Moderate levels of expression for seven transcripts were observed in leaves of sweetpotato at 72 h after imposition of salt stress (Fig 3). Interestingly, three transcripts (UN08712; SS4), UN06652; SS2), and UN85241; SS8)) were up-regulated in sweetpotato salt-stressed roots; however, UN08712 (SS4), UN90868 (SS5) and UN25202 (SS6) were not altered or were down-regulated in the leaves under salt stress at both 24 and 72 h of stress (Fig 3). Altogether, these results support the existence of mechanisms of salinity tolerance in beach morning glory, which appears to be partially conserved in sweetpotato. Expression analysis of five Na^+^/H^+^ transporters in the halophyte ice plant (*Mesembryanthemum crystallinum*) revealed a temporal correlation between salt accumulation and their expression levels in leaves, but not in roots [71]. On the other hand, *GmSALT3* gene of soybean, a moderately salt-sensitive crop, represents one of a few examples of that is preferentially expressed in root stelar cells and appears to be important for conferring whole plant salinity tolerance, because these cell types are already known to have a role in limiting salt transport to the shoot. Our results in sweetpotato and beach morning glory and other recent evidences indicate that different regulatory mechanisms are adopted by roots and shoots in response to salt stress [28]. SOS proteins may have novel roles in roots in addition to their functions in sodium homeostasis [28]. Novel genes involved in molecular responses to salt stress and for stress tolerance [4] have been reported in sweetpotato [4,34].This demonstrates that although novel salt tolerance responses are best studied in halophytes and species adapted to saline environments, researchers cannot overlook existing mechanisms of glycophytes. Indeed, most of our current knowledge on the molecular basis of salinity tolerance comes from studies of genes from salinity-sensitive model plant *Arabidopsis*. The results of the present work in both beach morning glory and sweetpotato suggest that the observed tissue-specificity in the expression pattern of certain genes warrants further detail characterization to identify their specific and coordinated roles in molecular and/or cellular mechanisms for plant’s adaptation to salinity.

### 3.3. *In silico* SSR analysis across multiple *Ipomoea* members

SSRs derived from coding regions are more conserved and their transferability has been proved to be high in plants. To further enhance the usefulness of the present beach morning glory transcriptome, an in silico analysis was performed to identify SSR markers. Six thousand two hundred thirty three transcripts were found harboring an SSR (S7 Table) and primers were designed for 4,897 SSRs (S8 Table). The remaining transcripts did not have enough sequences flanking the SSR motifs to design a primer. Mapping of the SSR primers in the beach morning glory transcriptome against the transcripts of four *Ipomoea* species (*I*. *batatas*, *I*. *nil*, *I*. *purpurea*, *I*. *trifida*) revealed that 434 primer pairs produced an amplicon in at least one transcript of one or more of the above species. Interestingly, these 434 SSR primer pairs were found targeting 1,221 regions in same number of transcripts across all *Ipomoea* species (S8 Table) and they represented a putative digital PCR product. Of these 1,221 amplicons, 627 were from sweetpotato, 460 from *I*. *purpurea*, 130 from *I*. *nil*, and 4 from *I*. *trifida*. Thus, these 434 SSRs cross-transferable and can be used in genetic studies of the *Ipomoea* genus. Two hundred eighty SSR primers matched with 627 sequences from the combined transcriptomes of sweetpotato (S9 and S10 Tables). Further, 219 out of 280 primers were polymorphic and produced a digital PCR fragment in sweetpotato that differed by at least 3-bp compared to the homologous product in beach morning glory. SSR markers have contributed to addressing the origins of sweetpotato [72] and to identification of loci involved in quantitative traits [73]. The identified cross-species transferable microsatellite markers from the present study might provide further insights into the origins of sweetpotato. The present work demonstrates the utility of an in silico approach in identifying SSRs among *Ipomoea* species in contrast to other similar studies that involved analysis of a single transcriptome [39,74]. These genic SSRs will be useful for genetic studies of sweetpotato and their relatives and to fill the gaps in the current AFLP markers-based genetic map of sweetpotato [75]. In addition, these cross-transferable genic markers will have their utility in existing interspecific crosses of sweetpotato and *I*. *trifida* to transfer valuable alleles.

## Conclusions

The present study reports the first reference transcriptome of beach morning glory, a sweetpotato relative possessing high level of salt-tolerance. The annotated transcripts represent a useful resource to unravel genes and pathways involved in salt stress tolerance in Convolvulacea members, since it revealed transcripts with similarities to genes of other plants known to be associated with salt stress responses. Expression profiling and comparative transcriptome analysis suggested similarities and differences between beach morning glory and sweetpotato. The knowledge and resources generated in this study in the form of novel genes/alleles and genic microsatellites may aid sweetpotato breeding programs to extend cultivation to saline soil environments. Future comparative characterization and functional validation of the identified candidate genes in beach morning glory vis-à-vis sweetpotato will further our understanding of the specifics of the salinity adaptation mechanisms in the halophyte relative of sweetpotato and subsequent translation to improve salinity tolerance in sweetpotato.

## Supporting Information

S1 Fig Growth of beach morning glory inside the greenhouse under control (upper panel) and salt stress (300 mM NaCl; lower panel). (PPTX)

S1 Table Summary of assembly functional annotation of sequences of beach morning glory. (XLSX)

S2 Table Summary of functional annotation of beach morning glory transcripts by selecting top BLAST hit in UniRef90. (XLSX)

S3 Table Summary of BLAST matching hits in TAIR10 database. E-value threshold was less or equal to 1E-06.
(XLSX)

S4 Table List of 4,045 beach morning glory transcripts selected from shoot and/or root tissues under salt stress. For further details please see S1 table.
(XLSX)

S5 Table Comparative analysis of beach morning glory transcripts by BLASTN against the transcriptome of four *Ipomoea* species. (XLSX)

S6 Table GO terms enrichment analysis by Fisher's Exact Test. (XLSX)

S7 Table List of unigenes from beach morning glory with detail of the SSR motif(s). (XLSX)

S8 Table List of beach morning glory SSR-containing sequences and corresponding primers. (XLSX)

S9 Table List of sequences of four *Ipomoea* species matching the SSRs-primer pairs from beach morning glory. (XLSX)

S10 Table Type and number of SSR motif and length of the repeats in beach morning glory transcriptome. (XLSX)

S1 Text De novo assembly output of beach morning glory reads in SAM format. (S1_Text_unigene.sam, ~200MB in size).
(ZIP)
