# Supplementary material for: Transcriptome Profiling of Beach Morning Glory (Ipomoea imperati) under Salinity and Its Comparative Analysis with Sweetpotato
Source: PLoS One. 2016 Feb 5;11(2):e0147398. doi: 10.1371/journal.pone.0147398 (PMC4743971; doi:10.1371/journal.pone.0147398)
Supplement: S1 Fig — (PPTX) [file pone.0147398.s001.pptx]

## Slide 1
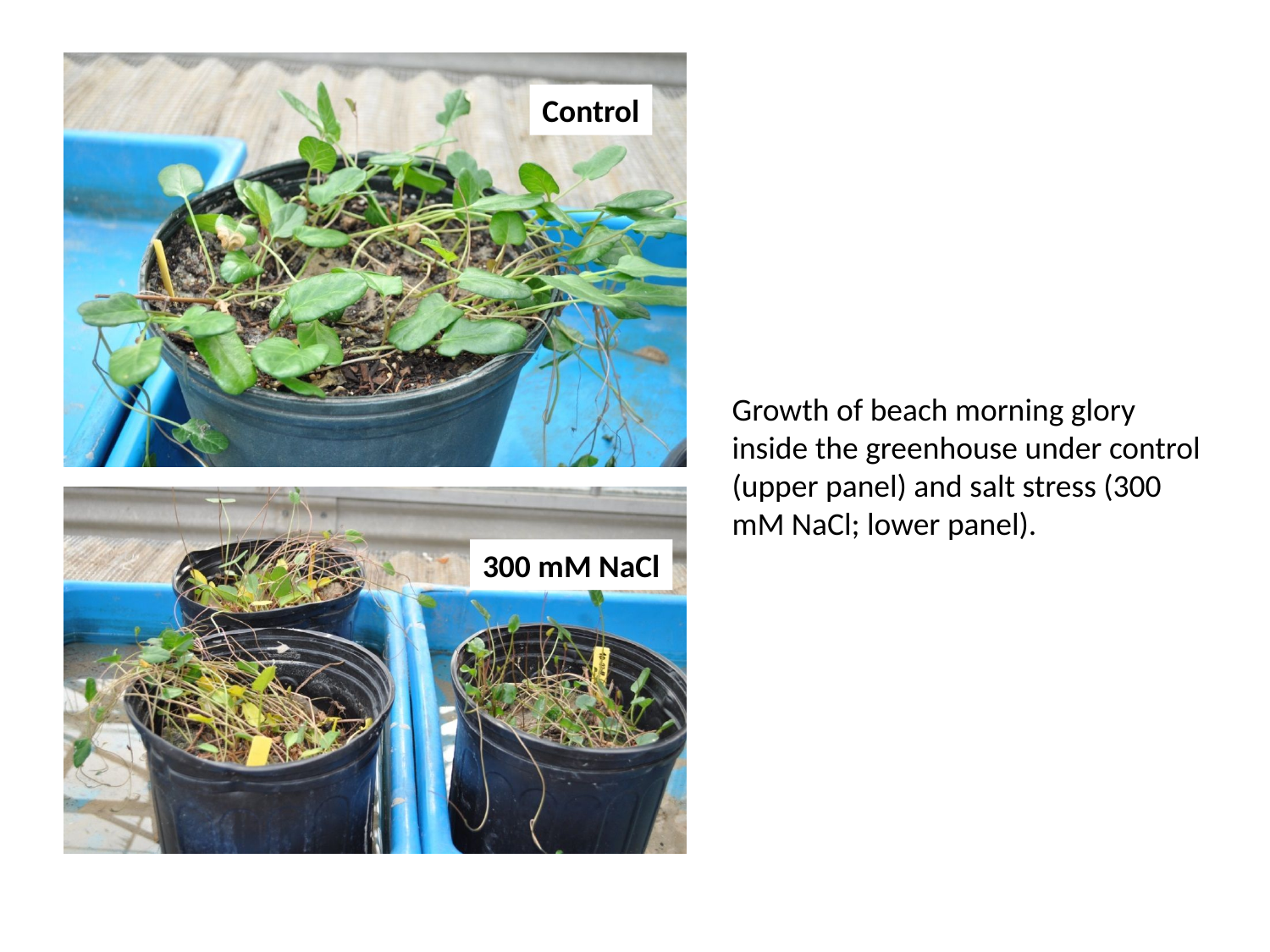

Control
Growth of beach morning glory
inside the greenhouse under control (upper panel) and salt stress (300 mM NaCl; lower panel).
300 mM NaCl
